# Insights into the Genetic Architecture of Early Stage Age-Related Macular Degeneration: A Genome-Wide Association Study Meta-Analysis

**DOI:** 10.1371/journal.pone.0053830

**Published:** 2013-01-11

**Authors:** Elizabeth G. Holliday, Albert V. Smith, Belinda K. Cornes, Gabriëlle H. S. Buitendijk, Richard A. Jensen, Xueling Sim, Thor Aspelund, Tin Aung, Paul N. Baird, Eric Boerwinkle, Ching Yu Cheng, Cornelia M. van Duijn, Gudny Eiriksdottir, Vilmundur Gudnason, Tamara Harris, Alex W. Hewitt, Michael Inouye, Fridbert Jonasson, Barbara E. K. Klein, Lenore Launer, Xiaohui Li, Gerald Liew, Thomas Lumley, Patrick McElduff, Barbara McKnight, Paul Mitchell, Bruce M. Psaty, Elena Rochtchina, Jerome I. Rotter, Rodney J. Scott, Wanting Tay, Kent Taylor, Yik Ying Teo, André G. Uitterlinden, Ananth Viswanathan, Sophia Xie, Wellcome Trust Case Control Consortium 2, Johannes R. Vingerling, Caroline C. W. Klaver, E. Shyong Tai, David Siscovick, Ronald Klein, Mary Frances Cotch, Tien Y. Wong, John Attia, Jie Jin Wang

**Affiliations:** 1 Centre for Clinical Epidemiology and Biostatistics, and School of Medicine and Public Health, University of Newcastle, Newcastle, New South Wales, Australia; 2 Icelandic Heart Association, Kopavogur, Iceland; 3 Faculty of Medicine, University of Iceland, Reykjavik, Iceland; 4 Singapore Eye Research Institute, Singapore National Eye Centre, Singapore, Singapore; 5 Department of Ophthalmology, Erasmus Medical Center, Rotterdam, The Netherlands; 6 Department of Epidemiology, Erasmus Medical Center, Rotterdam, The Netherlands; 7 Cardiovascular Health Research Unit, University of Washington, Seattle, Washington, United States of America; 8 Department of Medicine, University of Washington, Seattle, Washington, United States of America; 9 Centre for Molecular Epidemiology, National University of Singapore, Singapore, Singapore; 10 Center for Statistical Genetics, University of Michigan, Ann Arbor, Michigan, United States of America; 11 Centre for Eye Research Australia, University of Melbourne, Royal Victorian Eye and Ear Hospital, Melbourne, Australia; 12 Human Genetics Center and Human Genome Sequencing Center, University of Texas and Baylor College of Medicine, Houston, Texas, United States of America; 13 Department of Ophthalmology, Yong Loo Lin School of Medicine, National University of Singapore, Singapore, Singapore; 14 Saw Swee Hock School of Public Health, National University of Singapore, Singapore, Singapore; 15 Centre for Quantitative Medicine, Office of Clinical Sciences, Duke-NUS Graduate Medical School, Singapore, Singapore; 16 Laboratory of Epidemiology, Demography, and Biometry, National Institute on Aging, Intramural Research Program, National Institutes of Health, Bethesda, Maryland, United States of America; 17 Department of Pathology, University of Melbourne, Melbourne, Victoria, Australia; 18 Department of Ophthalmology, Landspitali University Hospital, Reykjavik, Iceland; 19 Department of Ophthalmology and Visual Science, School of Medicine and Public Health, University of Wisconsin, Madison, Wisconsin, United States of America; 20 Medical Genetics Institute, Cedars-Sinai Medical Center, Los Angeles, California, United States of America; 21 Centre for Vision Research, Department of Ophthalmology and the Westmead Millennium Institute, University of Sydney, Sydney, Australia; 22 Department of Statistics, University of Auckland, Auckland, New Zealand; 23 Department of Biostatistics, University of Washington, Seattle, Washington, United States of America; 24 Department of Epidemiology, University of Washington, Seattle, Washington, United States of America; 25 Department of Health Services, University of Washington, Seattle, Washington, United States of America; 26 Group Health Research Institute, Group Health Cooperative, Seattle, Washington, United States of America; 27 School of Biomedical Sciences, University of Newcastle, Newcastle, New South Wales, Australia and Hunter Medical Research Institute, and Hunter Area Pathology Service, Newcastle, New South Wales, Australia; 28 Department of Epidemiology and Public Health, National University of Singapore, Singapore, Singapore; 29 Department of Internal Medicine, Erasmus Medical Center, Rotterdam, The Netherlands; 30 Department of Clinical Chemistry, Erasmus Medical Center, Rotterdam, The Netherlands; 31 Moorfields Eye Hospital, London, United Kingdom; 32 Department of Medicine, National University of Singapore, Singapore, Singapore; 33 Duke-National University of Singapore Graduate Medical School, Singapore, Singapore; 34 Division of Epidemiology and Clinical Applications, National Eye Institute, Intramural Research Program, National Institutes of Health, Bethesda, Maryland, United States of America; 35 Department of Ophthalmology, National University of Singapore, Singapore, Singapore; 36 Department of Medicine, John Hunter Hospital and Hunter Medical Research Institute, Newcastle, New South Wales, Australia; Huazhong University of Science and Technology, China

## Abstract

Genetic factors explain a majority of risk variance for age-related macular degeneration (AMD). While genome-wide association studies (GWAS) for late AMD implicate genes in complement, inflammatory and lipid pathways, the genetic architecture of early AMD has been relatively under studied. We conducted a GWAS meta-analysis of early AMD, including 4,089 individuals with prevalent signs of early AMD (soft drusen and/or retinal pigment epithelial changes) and 20,453 individuals without these signs. For various published late AMD risk loci, we also compared effect sizes between early and late AMD using an additional 484 individuals with prevalent late AMD. GWAS meta-analysis confirmed previously reported association of variants at the complement factor H (*CFH*) (peak *P* = 1.5×10^−31^) and age-related maculopathy susceptibility 2 (*ARMS2*) (*P* = 4.3×10^−24^) loci, and suggested Apolipoprotein E (*ApoE*) polymorphisms (rs2075650; *P* = 1.1×10^−6^) associated with early AMD. Other possible loci that did not reach GWAS significance included variants in the zinc finger protein gene *GLI3* (rs2049622; *P* = 8.9×10^−6^) and upstream of *GLI2* (rs6721654; *P* = 6.5×10^−6^), encoding retinal Sonic hedgehog signalling regulators, and in the tyrosinase (*TYR*) gene (rs621313; *P* = 3.5×10^−6^), involved in melanin biosynthesis. For a range of published, late AMD risk loci, estimated effect sizes were significantly lower for early than late AMD. This study confirms the involvement of multiple established AMD risk variants in early AMD, but suggests weaker genetic effects on the risk of early AMD relative to late AMD. Several biological processes were suggested to be potentially specific for early AMD, including pathways regulating RPE cell melanin content and signalling pathways potentially involved in retinal regeneration, generating hypotheses for further investigation.

## Introduction

Age-related macular degeneration (AMD) is the most common cause of irreversible blindness in the elderly in many countries [1]. Clinically, the disease is believed to develop via a series of progressive stages [2]. Early AMD is characterised by abnormalities in the retinal pigment epithelium (RPE) and the deposition of small extracellular deposits, called drusen, between Bruch’s membrane and the RPE [3]. As the disease progresses, large drusen (>125 *µ*m in diameter) with or without fuzzy edges (soft indistinct or distinct drusen) may slowly disappear and be replaced by regions of retinal depigmentation (decreased pigment). Reticular drusen and large, soft drusen involving a large area of the macula are strong indicators of increased risk of progression to late, vision-impairing forms of this disease [4]. The two main phenotypes of late AMD are exudative AMD and geographic atrophy (GA). Exudative AMD is typified by sub-retinal neovascularisation with sensory retinal and/or RPE detachment, sub-retinal and/or sub-RPE haemorrhage followed by sub-retinal scarring. GA involves gradual degeneration and disappearance of RPE and photoreceptor cells within the macular area [3].

AMD is known to result from a complex interplay of multiple environmental and genetic factors. Disease risk increases strongly with age, with additional risk factors including smoking, possibly high body mass index, hypertension and cardiovascular disease [5], [6]. However, genetic factors have been shown to be strongly associated with AMD risk, with an increased genetic burden associated particularly with late forms of the disease [7]. For example, heritability estimates from twin studies are 0.45 for early AMD [8], but 0.71 for late AMD [9].

Case-control genome-wide association studies (GWAS) of late AMD have provided evidence for individual, large-effect risk variants in the *CFH*, *ARMS2*, *C2*/*CFB* and *C3* genes (estimated per allele odds ratios ranging from 1.7–4.5), and for additional, smaller-effect variants in *CFI*, *FRK*/*COL10A1*, *TNFRSF10A*, *LIPC*, *CETP*, *TIMP3*, *REST* and *VEGFA* (odds ratios ranging from 1.15–1.4) [10], [11], [12], [13], [14], [15]. Together with biological evidence, these findings highlight the importance of immune, inflammatory and lipid metabolic pathways in late AMD pathogenesis, while also implicating angiogenic, apoptotic, extracellular-matrix remodelling and melanosome trafficking processes [10], [11], [12], [13], [14], [15].

The vast majority of published GWAS have been conducted in samples of either exclusively or predominant late AMD cases (particularly late exudative AMD). This likely reflects the tendency for only symptomatic, late AMD cases to attend clinics, thus forming the majority of ascertained AMD samples available. In contrast, persons with early AMD are usually asymptomatic and less likely to be seen except in population-based studies. Therefore the genetic architecture of early AMD has been relatively under researched and is poorly understood [16].

Given the substantial heritability of AMD and the knowledge of modifiable risk factors that may reduce risk of progression to late, vision-impairing forms of this disease, improved understanding of the genetic architecture of early AMD may also be important. With this aim, we conducted a GWAS meta-analysis of early AMD, including approximately 4,000 well-characterised early AMD cases and 20,000 strictly defined controls without any drusen or with hard drusen only. Using a small set of mutually exclusive, late AMD cases, we also compared genetic effect sizes at validated AMD risk loci between early and late stages of the disease, to determine their relative importance for different disease stages. This study amalgamates a number of large population-based cohorts with GWAS data and AMD grading from retinal photographs.

## Materials and Methods

### Study Populations

The primary GWAS meta-analysis for early AMD was conducted in five European-ancestry cross-sectional cohorts (Table 1). These were recruited in the USA, Europe and Australia and contributed a total of 3,772 prevalent cases of early AMD and 16,033 contemporaneous controls from the Age, Gene/Environment Susceptibility-Reykjavik Study (AGES) [17], the Atherosclerosis Risk in Communities (ARIC) study [18], the Cardiovascular Health Study (CHS) [19], the Blue Mountains Eye Study (BMES) [20] and three distinct cohorts from the Rotterdam Study (RS) [21]: RS-I, RS-II and RS-III. In addition, two Asian-ancestry cross-sectional cohorts were included in secondary analyses; these included a total 264 prevalent early AMD cases and 3,926 controls from the Singapore Indian Eye Study (SINDI) [22] and the Singapore Malay Eye Study (SiMES) [23]. Subsequent candidate SNP meta-analyses for late AMD were performed in the European-ancestry cohorts including a total 498 prevalent cases of late AMD and 16,033 controls.

**Table 1:** Population characteristics for the individual cohorts.

	European Ancestry							Asian (Singapore) Ancestry	
	AGES	ARIC	BMES	CHS	RS-I	RS-II	RS-III	SiMES	SINDI
**Sample size**									
* Early AMD*	1031	399	796	258	1064	209	68	123	141
* Advanced AMD*	161	10	82	24	207	28	5	17	13
* No AMD*	1743	7717	1052	1501	1933	1076	1505	1965	1961
**Mean age (SD)**									
* Early AMD*	77.8 (5.3)	62.3 (5.5)	72.5 (9.0)	80.0 (4.7)	75.1 (7.7)	69.8 (9.0)	59.8 (8.0)	66.1 (10.0)	64.9 (10.6)
* Advanced AMD*	77.8 (5.3)	65.2 (5.6)	79.5 (9.2)	82.3 (4.6)	79.9 (7.0)	78.7 (9.1)	75.8 (10.9)	72.1 (7.1)	67.5 (10.4)
* No AMD*	74.9 (5.0)	60.1 (5.6)	66.3 (9.5)	78.4 (4.1)	74.9 (8.0)	66.9 (6.8)	55.7 (5.2)	58.2 (11.1)	56.8 (9.6)
**Female (%)**									
* Early AMD*	58.2	49.8	58.0	62.8	58.8	49.8	60.3	35.8	40.4
* Advanced AMD*	58.9	50.0	61.0	62.5	59.9	64.3	60.0	17.6	38.5
* No AMD*	56.6	53.2	55.7	61.9	56.2	49.8	56.2	54.2	50.2
**Current smokers (%)**									
* Early AMD*	12.6	16.6	7.6	7.4	20.7	25.4	29.4	17.4	10.6
* Advanced AMD*	12.8	20.0	14.0	4.2	27.0	18.5	20.0	47.1	7.7
* No AMD*	12.4	17.2	11.5	5.8	25.3	23.8	26.4	19.9	14.7

Each cohort obtained approval from relevant institutional review boards, and all participants provided written informed consent in accordance with the Declaration of Helsinki. All participating studies approved guidelines for collaboration, and a working group agreed, in advance, on phenotype definition, covariate selection and analytic plans for within-study analyses and meta-analyses of results. Details of each participating study are described below and in Tables S1, S2 in File S2.

### Phenotype Definitions

The same AMD phenotype definitions based on photographic grading were used for all the cohorts. Early AMD was defined as the presence of soft drusen (>63 µm) alone, retinal pigment epithelium (RPE) depigmentation alone or a combination of soft drusen with increased retinal pigment and/or depigmentation in the absence of late AMD. Late AMD was defined as the presence of exudative AMD or GA, as described in the International AMD classification [24]. Controls had no soft (distinct or indistinct) drusen or retinal pigment abnormalities (either depigmentation or increased pigment), and no signs of early or late AMD; controls were permitted to have hard drusen. Presence and severity of AMD lesions were assigned following the Wisconsin Age-Related Maculopathy grading system [25], based on masked assessment of fundus photographs. For the AGES, BMES, RS and Singapore-based cohorts, photographs were examined for both eyes using a retinal camera with pharmacological mydriasis, with cases satisfying AMD diagnostic criteria for at least one eye. For the ARIC and CHS cohorts, case and control diagnoses were based on examination of one, randomly selected eye using a retinal camera without mydriasis. Additional details are provided in Table S1 in File S2.

### Genotyping

The AGES and CHS samples were genotyped using the Illumina Human370 CNV quad array. The ARIC sample was genotyped using the Affymetrix SNP 6.0 GeneChip. The RS-I sample was genotyped using the Illumina Infinium II HumanHap550 chip. The RS-II, RS-III, SiMES and SINDI samples were genotyped using the Illumina Human610-Quad array. The BMES sample was genotyped using the Illumina Human670-Quad v1 custom array. All cohorts applied similar quality control (QC) procedures to genotype data prior to analysis. Briefly, this involved excluding SNPs with low call rate, pronounced deviation from Hardy-Weinberg equilibrium or low minor allele frequency, and individuals with low call rate, discrepant clinical and genotypic gender, evidence of cryptic relatedness based on IBS sharing (one member of each pair excluded) or outlying continental ancestry based on principal components analysis. The particular procedures and thresholds used for each study are detailed in Table S2 in File S2.

Following genotype quality control, all cohorts were imputed to approximately 2.5 million HapMap Phase II SNPs. European ancestry cohorts used the Caucasian (CEU) reference panel, and Asian ancestry cohorts used the combined Caucasian, Chinese, Japanese and African reference (CEU+CHB+JPT+YRI) panel. This provided data for a large set of overlapping SNPs for GWAS meta-analyses.

### Statistical Analysis

#### Primary meta-analysis of genetic associations with early AMD compared to controls

Individual European-ancestry cohorts conducted GWAS of early AMD. For each SNP, logistic regression was performed using a one-degree of freedom trend test assuming an additive effect of SNP allele dosage; logistic models included age, gender and the first two ancestry principal components as covariates. Regression coefficients and their standard errors were estimated to represent the change in log odds of AMD resulting from each additional copy of the test allele, after adjusting for specified covariates. Analyses were performed using the ProbABEL program [26] for AGES and ARIC cohorts, mach2dat [27] for BMES, R software for CHS and GRIMP software for the RS cohorts [28].

SNPs were included in meta-analyses if they exhibited an imputation quality score (defined as the ratio of observed to expected dosage variance) >0.3, minor allele frequency >0.01 and valid data in at least two cohorts. Strand data was available for each cohort and all results were synchronised to the forward strand. Fixed effects, inverse variance-weighted meta-analyses were performed using METAL software. The pre-specified threshold for genome-wide significant association was set at 5×10^−8^. SNPs not meeting this threshold but achieving *P*<1×10^−5^ were considered as suggestively associated; that is, potentially true signals, whose association may become more significant with increased sample size. Between-cohort heterogeneity of estimated SNP association with disease was assessed using *I^2^* and Cochran’s Q metrics. Quantile-Quantile (Q-Q) plots and Manhattan plots summarising the meta-analysis results were produced using R software. Regional association results were plotted using LocusZoom software [29]. Meta-analysis genomic control inflation factors (λ_GC_) were calculated as previously described [30], as were standardised values for a sample of 1000 cases and 1000 controls (λ_1000_) [30].

#### Comparison of effect sizes between early and advanced AMD for established AMD risk variants

In addition to the primary GWAS of early AMD, in European-ancestry cohorts, we performed association analyses for advanced AMD using a set of confirmed AMD-associated SNPs. Asian-ancestry cohorts had insufficient advanced AMD cases to perform these analyses. Confirmed AMD-associated SNPs were required to demonstrate genome-wide significant association (*P*<5×10^−8^) with advanced AMD in a published report, and were identified using the NIH National Human Genome Research Institute (NHGRI) Catalog of Published Genome-Wide Association Studies with search terms “Age-related macular degeneration” and “Age-related macular degeneration (wet)”. The identity and association evidence for SNPs returned by the NHGRI search were corroborated with original published reports in each case. In turn, the original reports were checked for appropriate AMD phenotype definition and searched to ensure no SNPs meeting eligibility criteria had been omitted from the NHGRI catalog. Statistical analyses of individual cohorts and meta-analyses of estimated SNP effect sizes were performed as described above.

For the selected set of confirmed AMD-associated SNPs, estimated SNP effect sizes for late and early AMD were statistically compared using summary effect estimates from the European-ancestry meta-analyses. The difference between the regression coefficients was assessed using a two-tailed *Z* test [31] formulated as
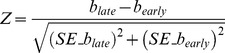
where 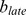, 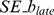 and 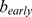, 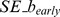 represent the meta-analysis summary effect estimates and their standard errors for late and early AMD, respectively. A total of 21 SNPs were assessed for effect size differences. Each test was performed at a pre-specified significance level of 0.00238, corresponding to a family-wise type I error rate of 0.05 after Bonferroni adjustment. We note that this threshold is somewhat conservative, as multiple, correlated SNPs were included from the *CFH* and *ARMS2* loci.

### Secondary, Trans-ethnic Meta-analysis of Genetic Associations with Early AMD

To extend on the results of the European-ancestry analysis, a secondary meta-analysis was conducted including all European-ancestry and Singapore-based, Asian-ancestry cohorts. Analyses in individual cohorts were performed via logistic regression as described for the primary European analysis. Fixed effects, inverse variance-weighted meta-analyses including all European (AGES, ARIC, BMES, CHS and RS) and Singapore-based (SiMES and SINDI) cohorts were performed using METAL. For a set of selected, validated AMD-associated variants (described above), we also tabulated ancestry-specific effect sizes separately for European- and Asian-ancestry cohorts.

## Results

### Primary Meta-analysis of Genetic Associations with Early AMD

For each of the individual cohorts, summary demographic characteristics are shown by AMD status in Table 1. A Q-Q plot summarising the distribution of all *P*-values showed excellent agreement with the expected null distribution throughout all but the extreme tail of the distribution (Figures S2 and S5a in File S1). The overall meta-analysis genomic control inflation factor (λ_GC_ = 1.021) indicated an absence of confounding by population stratification or other artefacts, as did the scaled, standardised value (λ_1000_ = 1.003).

The primary meta-analysis detected highly significant association of numerous SNPs within *CFH* and *ARMS2/HTRA1* loci with risk of early AMD (Table 2, Table S4 in File S3, Figure S1 in File S1). The peak results at these loci were detected for the *CFH* SNP rs1329424 (OR[T] = 1.41, 95% CI: 1.33–1.50, *P*-value = 1.5×10^−31^) and the *ARMS2/HTRA1* SNP rs3793917 (OR[G] = 1.43, 95% CI: 1.34–1.54, *P*-value = 4.3×10^−24^).

**Table 2:** Results for SNPs showing suggestive evidence of association (*P*<1×10^−5^) in the primary (European-ancestry) meta-analysis of early AMD.

Chr	BP^a^	SNP	EA^b^	Freq^c^	OR (95% CI)^d^	*P* e	*I^2f^*	Het*P* g	Gene Locus	Nearby Genes^h^
1	175,835,422	rs16851585	c	0.92	0.77 (0.69, 0.86)	5.0E-06	63.8	0.011	intergenic	
1	194,912,799	rs1329424	t	0.38	1.41 (1.33, 1.49)	1.5E-31	26	0.230	*CFH*	
1	206,106,094	rs1967689	c	0.25	0.85 (0.8, 0.91)	5.1E-06	36.6	0.149	intergenic	*CD34*, *CD46*
2	121,018,381	rs6721654	t	0.08	1.26 (1.14, 1.4)	6.5E-06	0	0.511	intergenic	*GLI2*, *INHBB*
4	117,143,633	rs17586843	t	0.78	1.18 (1.1, 1.27)	1.5E-06	0	0.817	intergenic	
6	106,366,821	rs7750345	a	0.75	1.16 (1.09, 1.24)	6.8E-06	57.1	0.030	intergenic	
7	42,142,807	rs2049622	a	0.49	0.87 (0.83, 0.93)	8.9E-06	0	0.779	*GLI3*	
8	127,401,839	rs11986011	t	0.02	2.5 (1.68, 3.71)	5.0E-06	49.3	0.095	intergenic	*FAM84B*
10	54,245,002	rs6480975	c	0.84	1.21 (1.12, 1.32)	2.8E-06	69.6	0.003	intergenic	*MBL2*
10	124,209,265	rs3793917	c	0.80	0.69 (0.64, 0.74)	4.3E-24	34.8	0.162	*ARMS2/HTRA1*	
11	82,499,030	rs4293143	t	0.69	0.85 (0.79, 0.91)	7.8E-06	0	0.774	intergenic	*PCF11*, *RAB30*
11	88,553,311	rs621313	a	0.51	0.87 (0.83, 0.92)	3.5E-06	49.9	0.063	*TYR*	
13	36,963,446	rs9646096	a	0.95	0.74 (0.65, 0.84)	6.0E-06	0	0.604	intergenic	*POSTN*, *TRPC4*
19	3,895,240	rs10406174	a	0.11	1.24 (1.13, 1.36)	5.6E-06	58.7	0.034	intergenic	*ITGB1BP3*, *DAPK3*
19	50,084,094	rs6857	t	0.15	0.81 (0.74, 0.88)	1.4E-06	25.8	0.232	*PVRL2*	
19	50,087,459	rs2075650	a	0.86	1.23 (1.13, 1.34)	1.1E-06	7.2	0.373	*APOE/TOMM40*	

Where multiple correlated SNPs in the same gene/region showed similar association evidence, the most strongly associated SNP is shown.

a NCBI Human Genome Build 36.3 coordinates;

b Effective allele;

c Frequency of the effective allele;

d Estimated odds ratio and 95% confidence interval for the effect of each additional copy of the effective allele, based on the fixed-effects, inverse variance-weighted meta-analysis of European-ancestry cohorts;

e *P*-value associated with the estimated OR;

f Heterogeneity *I^2^* statistic;

g Heterogeneity *P*-value, based on Cochran’s Q statistic;

h within a 500 kb genomic region centred on the associated SNP.

**Figure 1:**
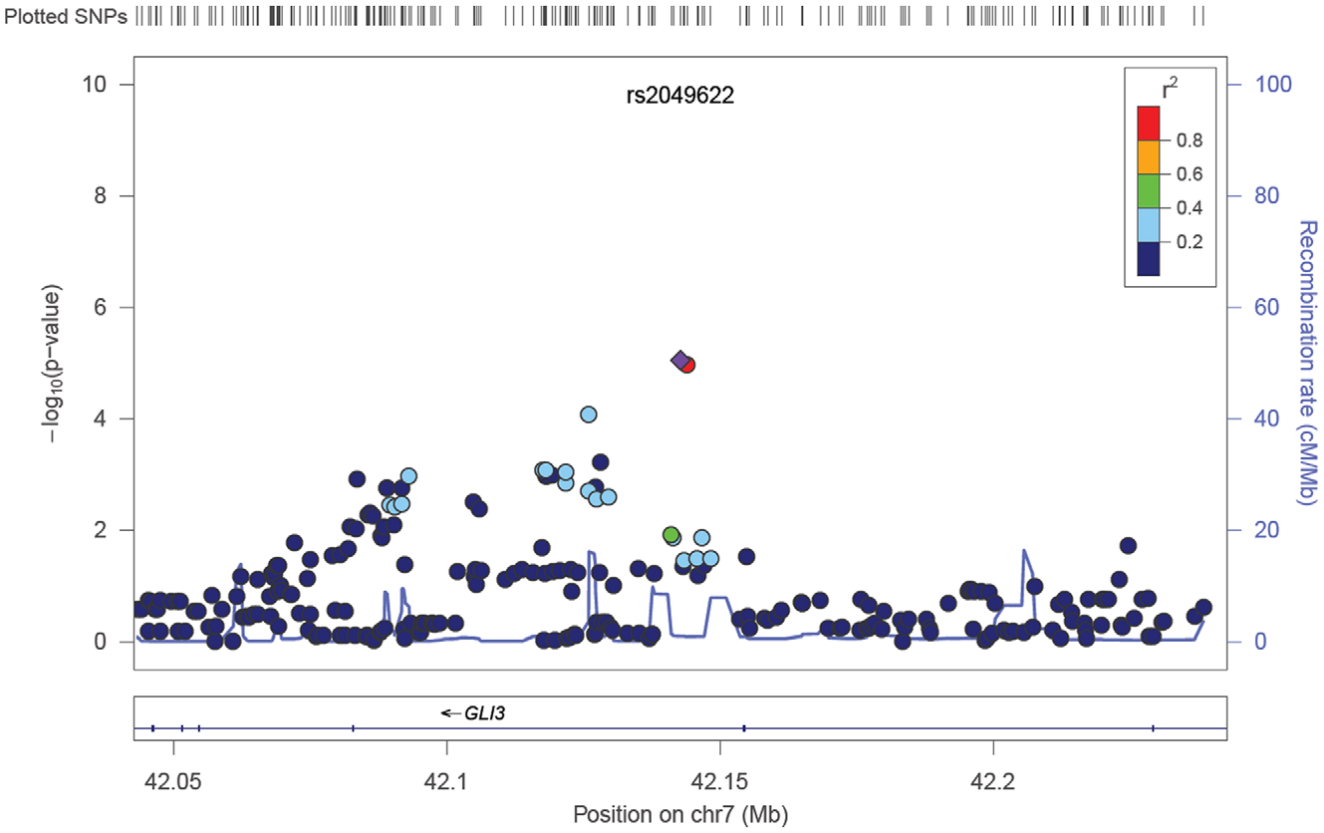
Regional association results for chromosome 7 SNPs in the *GLI*-Kruppel family member *GLI3* gene. The index, associated SNP is named and shown as a purple diamond (rs2049622: *P* = 8.9×10^−6^); remaining SNPs are colored according to the strength of LD (*r^2^*) with the index SNP (see figure legend). Pairwise LD and local recombination rates were calculated using HapMap CEU population data (Phase 2, release #22), with annotated genes mapped according to NCBI Build 36 sequence position.

**Figure 2:**
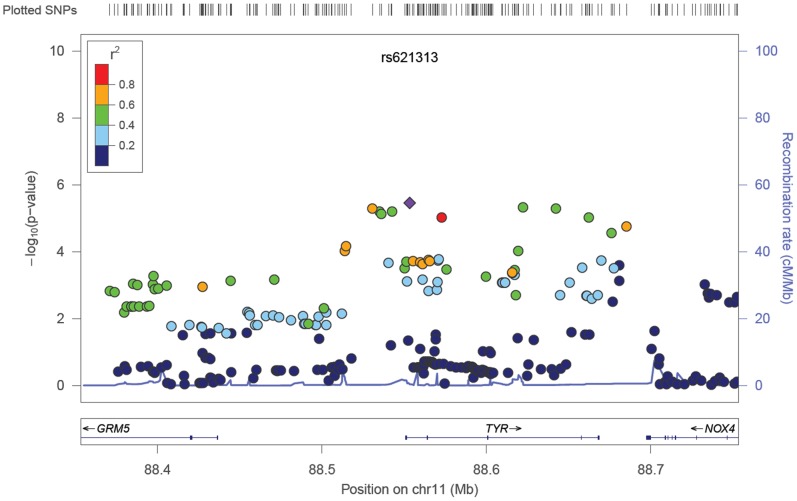
Regional association results for chromosome 11 SNPs in the tyrosinase precursor (*TYR*) gene. The index, associated SNP is named and shown as a purple diamond (rs621313: *P* = 3.5×10^−6^).

**Figure 3:**
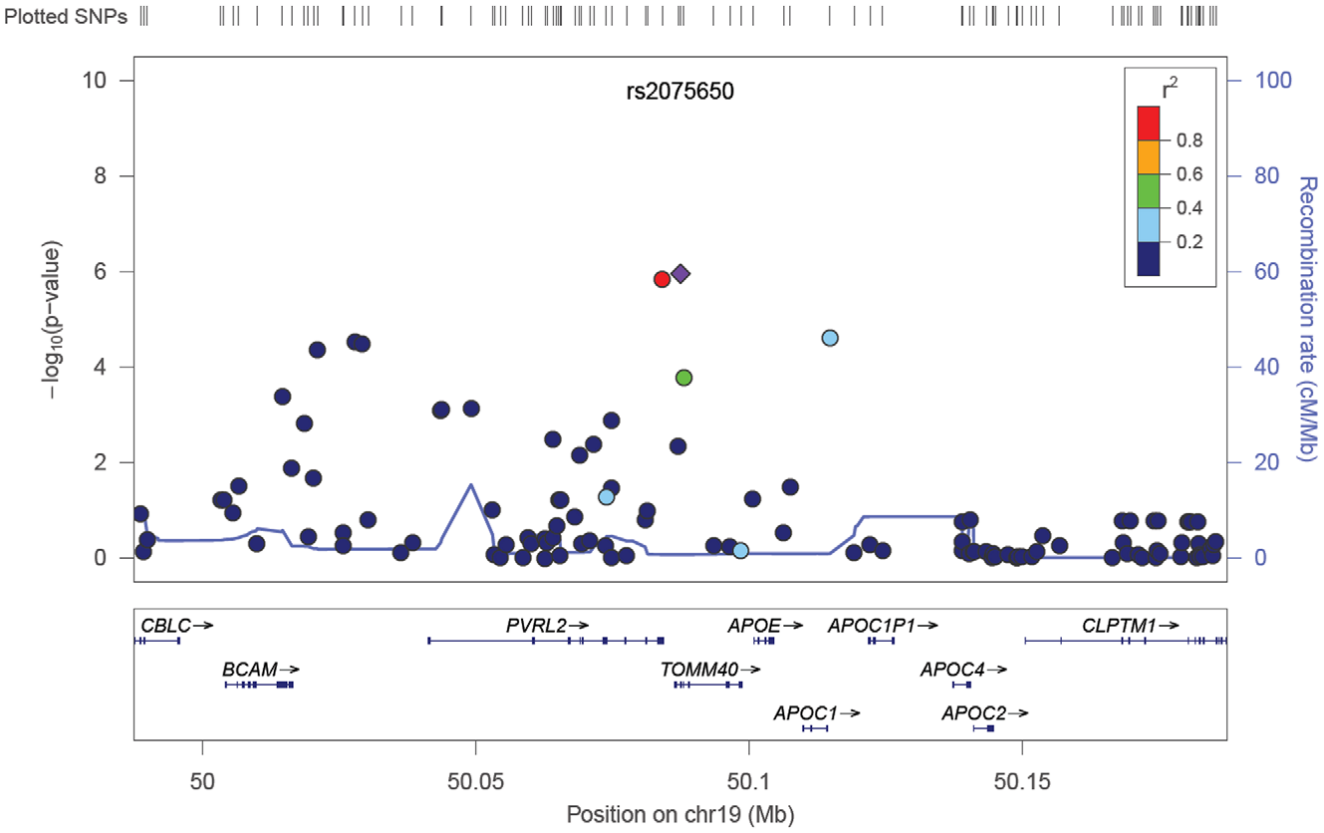
Regional association results for chromosome 19 SNPs in the *PVRL2*/*APOE*/*TOMM40* gene cluster. The index, associated SNP is named and shown as a purple diamond (rs2075650: *P* = 1.1×10^−6^).

Aside from SNPs at the *CFH* and *ARMS/HTRA1* loci, no additional variants reached genome-wide significance. However, a number of confirmed and plausible candidate genes contained SNPs demonstrating possible association with early AMD (5×10^−8^<*P*<1×10^−5^) (Table 2 and Table S5 in File S3). These included SNPs in the *ApoE*/*TOMM40* gene cluster on chromosome 19, the *GLI3* gene on chromosome 7 and the tyrosinase gene (*TYR*) on chromosome 11 (see Figures 1, 2, 3), and SNPs in the vicinity of the complement-related genes *CD46* and *MBL2*.

### Comparison of Effect Sizes between Early and Late AMD for Established AMD Risk Variants

SNPs in genes *C2/CFB*, *C3*, *CFI*, *CETP*, *TIMP3*, *TNFRSF10A*, *FRK/COL10A1*, *REST* and *LIPC* (*upstream*) that have previously demonstrated genome-wide significance with advanced AMD showed only modest association with early AMD (Table 3). Of these published associated variants, the strongest associations with early AMD were observed for rs429608 in *C2/CFB* (OR[G] = 1.18, 95% CI: 1.08–1.27, *P*-value = 9.6×10^−5^), rs2230199 in *C3* (OR[C] = 1.18, 95% CI: 1.08–1.29, *P*-value = 2.5×10^−4^) and rs13278062 in *TNFRSF10A* (OR[T] = 1.11, 95% CI: 1.05–1.18, *P*-value = 5×10^−4^). For many published SNPs we detected some evidence of association with both late and early AMD (at α = 0.05), observing similar odds ratios for late AMD as those previously reported (Table 3). Notably, for most of these SNPs the estimated effect size for late AMD was several-fold higher than that for early AMD. The absolute fold-change was ∼3-4-fold for risk variants in *CFH*, ∼3-fold for variants in *ARMS2/HTRA1*, ∼3-6-fold for variants in *C2/CFB*, ∼4-fold for a SNP in *CETP* and ∼2-fold for variants in *C3* and *FRK/COL10A1*. For SNPs in *CFH*, *ARMS2/HTRA1*, and *C2/CFB*, the heterogeneity of effect size between early and late AMD was statistically significant after multiple testing adjustment (*P*<0.05). The only SNP showing some evidence for association but a *weaker* effect for late AMD was rs10033900 in *CFI*, which showed very marginal association (*P* = 0.038) with early AMD but no association with late AMD, and had a slightly higher estimated effect for early than late AMD (1.8-fold greater); however the difference in effect size was not statistical significant (*P*>0.05). Previously published AMD risk variants in *REST*, *LIPC* upstream and *TIMP3* showed no evidence of association (at α = 0.05) with either late or early AMD in our study.

**Table 3:** Comparison of estimated effect sizes for early versus advanced AMD for published SNPs showing genome-wide significant association with AMD.

						Early AMD				Advanced AMD					
SNP	Nearby genes^a^	Chr	BP^b^	EA^c^	Freq^d^	Beta^e^	OR (95% CI)^f^	*P* g	*P*_het_ h	Beta	OR (95% CI)	*P*	*P*_het_	*P*_het_ i (adv vs early)	Fold change^j^
rs1329424	*CFH* 1	1	194,912,799	T	0.37	0.35	1.41 (1.33, 1.50)	1.5E-31	0.23	1.09	2.99 (2.56, 3.48)	1.6E-44	0.16	**<1E-16**	3.17
rs1061170	*CFH* 2	1	194,925,860	C	0.34	0.39	1.47 (1.37, 1.57)	2.4E-28	0.18	1.10	3.02 (2.56, 3.56)	2.3E-39	0.01	**2.8E-15**	2.87
rs10737680	*CFH* 1	1	194,946,078	A	0.58	0.31	1.36 (1.28, 1.44)	8.7E-26	0.01	1.24	3.45 (2.9, 4.1)	2.9E-45	0.81	**<1E-16**	4.03
rs1410996	*CFH* 2	1	194,963,556	G	0.58	0.31	1.36 (1.28, 1.44)	1.1E-25	0.01	1.23	3.43 (2.89, 4.07)	4.0E-45	0.82	**<1E-16**	4.03
rs380390	*CFH* 3	1	194,967,674	C	0.59	0.33	1.39 (1.31, 1.47)	5.5E-30	0.22	1.05	2.85 (2.45, 3.32)	3.3E-41	0.11	**<1E-16**	3.18
rs1329428	*CFH* 4	1	194,969,433	C	0.58	0.30	1.36 (1.28, 1.44)	2.1E-25	0.02	1.22	3.40 (2.86, 4.03)	1.8E-44	0.82	**<1E-16**	4.02
rs1713985	*REST* 5	4	57,481,207	G	0.09	0.08	0.92 (0.84, 1.02)	0.111	0.004	0.04	0.97 (0.76, 1.23)	0.770	0.65	1	0.46
rs10033900	*CFI* 2	4	110,878,516	T	0.49	0.06	1.06 (1.00, 1.12)	0.038	0.06	0.03	1.03 (0.90, 1.19)	0.639	0.76	1	0.56
rs9332739	*C2* 2	6	32,011,783	G	0.94	0.15	0.86 (0.75, 0.99)	0.030	0.32	0.73	0.48 (0.33, 0.72)	3.2E-04	0.73	7E-03	4.83
rs9380272	*C2* 1	6	32,013,989	A	0.01	0.08	1.09 (0.73, 1.62)	0.678	0.6	0.47	1.6 (0.87, 2.94)	0.130	0.84	0.298	5.57
rs641153	*CFB* 2	6	32,022,159	G	0.90	0.17	0.84 (0.76, 0.93)	1.1E-03	0.35	0.58	0.56 (0.42, 0.75)	7.9E-05	0.65	8.4E-03	3.43
rs429608	*C2/CFB* 1	6	32,038,441	G	0.85	0.16	1.18 (1.08, 1.27)	9.6E-05	0.51	0.62	1.87 (1.48, 2.35)	1.1E-07	0.54	**2.0E-04**	3.87
rs1999930	*FRK/COL10A1* 2	6	116,493,827	C	0.72	0.10	0.91 (0.85, 0.96)	1.7E-03	0.86	0.21	0.81 (0.7, 0.95)	0.010	0.78	0.220	2.06
rs13278062	*TNFRSF10A* 5	8	23,138,916	T	0.51	0.11	1.11 (1.05, 1.18)	5.0E-04	0.65	0.13	1.14 (0.99, 1.32)	0.076	0.99	0.752	1.24
rs10490924	*ARMS2* 2	10	124,204,438	T	0.20	0.36	1.43 (1.33, 1.54)	9.1E-24	0.16	1.10	2.99 (2.54, 3.53)	5.5E-39	0.14	**6.6E-16**	3.06
rs3793917	*ARMS2* 1	10	124,209,265	G	0.20	0.36	1.43 (1.34, 1.54)	4.3E-24	0.16	1.09	2.98 (2.53, 3.51)	6.7E-39	0.13	**8.8E-16**	3.03
rs10468017	*LIPC upstr* 6	15	56,465,804	C	0.70	0.06	0.95 (0.89, 1.01)	0.083	0.12	0.02	0.98 (0.84, 1.15)	0.828	0.49	1	0.32
rs493258	*LIPC upstr* 6	15	56,475,172	C	0.53	0.02	0.98 (0.93, 1.04)	0.581	0.04	0.02	0.98 (0.85, 1.13)	0.829	0.79	1	0.98
rs3764261	*CETP* 1	16	55,550,825	A	0.33	0.07	1.07 (1.01, 1.14)	0.021	0.94	0.27	1.31 (1.13, 1.51)	2.8E-04	0.19	0.0138	3.77
rs2230199	*C3* 1	19	6,669,387	C	0.19	0.16	1.18 (1.08, 1.29)	2.5E-04	0.08	0.37	1.44 (1.18, 1.77)	4.0E-04	0.78	0.072	2.24
rs9621532	*TIMP3* 1	22	31,414,511	A	0.94	0.05	1.05 (0.92, 1.20)	0.445	0.16	0.08	1.09 (0.78, 1.52)	0.627	0.94	0.866	1.60

a Superscript shows reference for the largest study reporting genome-wide association of the relevant SNP with AMD:

1 Chen et al, 2010 ^11^.

2 Yu et al, 2011 ^15^.

3 Klein et al, 2005 ^12^.

4 Kopplin et al, 2010 ^13^.

5 Arakawa et al, 2011 ^10^.

6 Neale et al, 2010 ^14^.

b NCBI Human Genome Build 36.3 coordinates;

c Effective allele;

d Frequency of the effective allele;

e Summary meta-analysis regression coefficient, indicating the overall, estimated change in log(odds) associated with each additional copy of the effective allele;

f Estimated odds ratio and 95% confidence interval for each additional copy of the effective allele, based on fixed-effects meta-analysis of European-ancestry cohorts;

g *P*-value associated with the estimated OR;

h Heterogeneity *P*-value, based on Cochran’s Q statistic;

i *P*-value from test of heterogeneity of regression coefficients between early and advanced AMD. The threshold for study-wise significance was 0.0024, after accounting for multiple tests. Significant results are shown in bold;

j Ratio of regression coefficient for advanced vs early AMD, formulated as Beta_adv_/Beta_early._

*Notes:* This study did not have data and could not assess association for additional published SNPs rs4711751 in *VEGFA* and rs11200638 in *HTRA1*.

Due to the limited number of late AMD cases, our direct, internal comparison provided modest power to detect effect size differences between early and late AMD. Hence, we repeated the effect size comparisons for validated AMD-associated SNPs, using published effect estimates from well-powered GWAS of late AMD. Table 4 shows analogous comparisons as shown Table 3, with late AMD effect estimates replaced with published effect estimates. This analysis produced largely consistent results as the internal comparison. However, in addition to SNPs in *CFH*, *ARMS2*/*HTRA1* and *C2*/*CFB* numerous additional SNPs showed significant effect size differences between early and late AMD – including SNPs in *REST*, *TNFRSF10A*, *C3*, *TIMP3* and upstream of *LIPC*.

**Table 4:** Comparison of effect sizes for early AMD from this study versus published effect estimates for late AMD.

						Early AMD – this study			Late AMD –published data				
SNP	Nearbygenes^a^	Chr	BP^b^	EA^c^	Freq^d^	Beta^e^	OR (95% CI)^f^	*P* g	Beta	OR (95% CI)^h^	*P*	*P*_het_ (adv vs early)^i^	Fold change^j^
rs1329424	*CFH* 1	1	194,912,799	t	0.37	0.35	1.41 (1.33, 1.50)	1.5E-31	0.63	1.88 (1.68, 2.10)	6.40E-16	**2.46E-06**	1.83
rs1061170	*CFH* 2	1	194,925,860	C	0.34	0.39	1.47 (1.37, 1.57)	2.4E-28	0.88	2.41 (NR)	1.30E-261	–	2.28
rs10737680	*CFH* 1	1	194,946,078	a	0.58	0.31	1.36 (1.28, 1.44)	8.7E-26	1.13	3.11 (2.76, 3.51)	1.60E-76	**<1E-16**	3.70
rs1410996	*CFH* 2	1	194,963,556	G	0.58	0.31	1.36 (1.28, 1.44)	1.1E-25	1.00	2.71 (NR)	7.40E-235	–	3.26
rs380390	*CFH* 3	1	194,967,674	c	0.59	0.33	1.39 (1.31, 1.47)	5.5E-30	1.53	4.6 (2.0, 11)	4.10E-08	**1.24E-03**	4.63
rs1329428	*CFH* 4	1	194,969,433	C	0.58	0.30	1.36 (1.28, 1.44)	2.1E-25	1.02	2.78 (NR)	1.90E-52	–	3.36
rs1713985	*REST* 5	4	57,481,207	G	0.09	0.08	0.92 (0.84, 1.02)	0.111	0.26	1.3 (1.19, 1.42)	2.34E-08	**1.40E-03**	3.36
rs10033900	*CFI* 2	4	110,878,516	t	0.49	0.06	1.06 (1.00, 1.12)	0.038	0.17	1.18 (NR)	4.10E-10	–	2.81
rs9332739	*C2* 2	6	32,011,783	G	0.94	0.15	0.86 (0.75, 0.99)	0.030	0.78	2.17 (NR)	2.40E-23	–	5.17
rs9380272	*C2* 1	6	32,013,989	a	0.01	0.08	1.09 (0.73, 1.62)	0.678	1.46	4.31 (2.76, 6.87)	2.30E-08	**1.61E-06**	17.31
rs641153	*CFB* 2	6	32,022,159	G	0.90	0.17	0.84 (0.76, 0.93)	1.1E-03	0.62	1.85 (NR)	5.50E-31	–	3.63
rs429608	*C2/CFB* 1	6	32,038,441	G	0.85	0.16	1.18 (1.08, 1.27)	9.6E-05	0.77	2.16 (1.84, 2.53)	2.50E-21	**7.89E-12**	4.77
rs1999930	*FRK/COL10A1* 2	6	116,493,827	C	0.72	0.10	0.91 (0.85, 0.96)	1.7E-03	0.14	1.15 (1.10, 1.20)	1.10E-08	7.76E-02	1.40
rs13278062	*TNFRSF10A* 5	8	23,138,916	t	0.51	0.11	1.11 (1.05, 1.18)	5.0E-04	0.31	1.37 (1.25, 1.49)	1.03E-12	**4.32E-05**	2.98
rs10490924	*ARMS2* 2	10	124,204,438	t	0.20	0.36	1.43 (1.33, 1.54)	9.1E-24	1.08	2.94 (NR)	3.6E-322	–	3.01
rs3793917	*ARMS2* 1	10	124,209,265	G	0.20	0.36	1.43 (1.34, 1.54)	4.3E-24	1.22	3.40 (2.94, 3.94)	4.10E-60	**<1E-16**	3.39
rs10468017	*LIPC upstr* 6	15	56,465,804	C	0.70	0.06	0.95 (0.89, 1.01)	0.083	0.20	1.22 (1.15, 1.30)	1.34E-08	**3.35E-04**	3.51
rs493258	*LIPC upstr* 6	15	56,475,172	C	0.53	0.02	0.98 (0.93, 1.04)	0.581	0.15	1.16 (1.11, 1.22)	1.61E-08	**7.67E-05**	9.49
rs3764261	*CETP* 1	16	55,550,825	a	0.33	0.07	1.07 (1.01, 1.14)	0.021	0.17	1.19 (1.12, 1.27)	7.40E-07	4.77E-03	2.44
rs2230199	*C3* 1	19	6,669,387	c	0.19	0.16	1.18 (1.08, 1.29)	2.5E-04	0.55	1.74 (1.47, 2.06)	1.00E-10	**1.48E-05**	3.37
rs9621532	*TIMP3* 1	22	31,414,511	a	0.94	0.05	1.05 (0.92, 1.20)	0.445	0.34	1.41 (1.27, 1.57)	1.10E-11	**1.74E-04**	6.66

a Superscript shows reference for the largest study reporting genome-wide association of the relevant SNP with late AMD, from which the “Late AMD” effect estimates were derived:

1 Chen et al, 2010 ^11^.

2 Yu et al, 2011 ^15^.

3 Klein et al, 2005 ^12^.

4 Kopplin et al, 2010 ^13^.

5 Arakawa et al, 2011 ^10^.

6 Neale et al, 2010 ^14^.

b NCBI Human Genome Build 36.3 coordinates;

c Effective allele;

d Frequency of the effective allele;

e Summary meta-analysis regression coefficient, indicating the overall, estimated change in log(odds) associated with each additional copy of the effective allele;

f Estimated odds ratio and 95% confidence interval for each additional copy of the effective allele, based on fixed-effects meta-analysis of European-ancestry cohorts;

g *P*-value associated with the estimated OR;

h NR: not reported;

i *P*-value from test of heterogeneity of regression coefficients between early and advanced AMD. The threshold for study-wise significance was 0.0036, after accounting for multiple tests. Significant results are shown in bold. Heterogeneity could not be assessed for SNPs with no published confidence interval for the late AMD effect estimate;

j Ratio of regression coefficient for advanced vs early AMD, formulated as Beta_adv_/Beta_early_.

*Notes:* This study did not have data and could not assess association for additional published SNPs rs4711751 in *VEGFA* and rs11200638 in *HTRA1*.

### Secondary, Trans-ethnic Meta-analysis of Genetic Associations with Early AMD

Secondary meta-analysis including all European samples plus the two Asian-ancestry samples produced very similar results to the primary analysis. There was no evidence of systematic bias of test statistics (Figure S4 in File S1; λ_GC_ = 1.022, λ_1000_ = 1.003) and SNPs in the *CFH* and *ARMS2/HTRA1* loci reached genome-wide significance (Figure S3 in File S1). SNPs in the vicinity of *ApoE* achieved slightly greater significance (peak *P* = 9.7×10^−7^ at rs6857), while the peak evidence for *TYR* (*P* = 9×10^−6^ at rs10830228) and *GLI3* (*P* = 1×10^−5^ at rs2049622) was slightly reduced. Several additional intergenic regions were also identified at P<10^−5^ (Table S6 in File S3).

For the set of selected, validated AMD-associated variants (described above), ancestry-specific effect sizes are shown separately for European and Asian ancestry cohorts in Table 5. Considering the low power of the Singapore-based analysis – with <300 early AMD cases represented across the two constituent samples – the effect sizes in the European and Asian-ancestry cohorts were largely consistent for the candidate SNPs, with confidence intervals for effect sizes overlapping for 8 of the 16 assessed SNPs. The principal exception was for the 6 SNPs located within the *CFH* gene, which showed strong association in the European analysis, but no association in our small Singapore-based analysis.

**Table 5:** Tabulation of estimated effect sizes for European-ancestry versus Asian ancestry analyses of early AMD, for the validated AMD-associated SNPs shown in Table 3.

					European Early AMD					Singapore-based Asian Early AMD				
SNP	Nearby genes	chr	BP	EA	Freq^a^	Beta	OR (95% CI)	*P*	*P*_het_	Freq^b^	Beta	OR (95% CI)	*P*	*P*_het_
rs1329424	*CFH*	1	194,912,799	t	0.37	0.35	1.41 (1.33, 1.50)	1.5E-31	0.23	0.25	−0.01	0.99 (0.84, 1.16)	9.46E-01	0.1523
rs1061170	*CFH*	1	194,925,860	C	0.34	0.39	1.47 (1.37, 1.57)	2.4E-28	0.18	0.22	0.08	1.07 (0.88, 1.31)	4.58E-01	0.0654
rs10737680	*CFH*	1	194,946,078	a	0.58	0.31	1.36 (1.28, 1.44)	8.7E-26	0.01	0.48	−0.03	0.97 (0.88, 1.06)	5.27E-01	0.3198
rs1410996	*CFH*	1	194,963,556	G	0.58	0.31	1.36 (1.28, 1.44)	1.1E-25	0.01	0.48	−0.03	0.97 (0.89, 1.06)	5.49E-01	0.3333
rs380390	*CFH*	1	194,967,674	c	0.59	0.33	1.39 (1.31, 1.47)	5.5E-30	0.22	0.75	0.02	1.02 (0.87, 1.19)	7.80E-01	0.2013
rs1329428	*CFH*	1	194,969,433	C	0.58	0.30	1.36 (1.28, 1.44)	2.1E-25	0.02	0.47	−0.04	0.96 (0.88, 1.05)	4.11E-01	0.4216
rs1713985	*REST*	4	57,481,207	G	0.09	0.08	0.92 (0.84, 1.02)	0.111	0.004	0.30	0.01	1 (0.9, 1.12)	9.13E-01	0.2978
rs10033900	*CFI*	4	110,878,516	t	0.49	0.06	1.06 (1.00, 1.12)	0.038	0.06	0.67	−0.06	0.93 (0.84, 1.04)	2.32E-01	0.3802
rs429608	*C2/CFB*	6	32,038,441	G	0.85	0.16	1.18 (1.08, 1.27)	9.6E-05	0.51	0.78	0.01	1.01 (0.85, 1.2)	8.94E-01	0.2462
rs13278062	*TNFRSF10A*	8	23,138,916	t	0.51	0.11	1.11 (1.05, 1.18)	5.0E-04	0.65	0.51	0.08	1.07 (0.98, 1.18)	1.13E-01	0.09459
rs10490924	*ARMS2*	10	124,204,438	t	0.20	0.36	1.43 (1.33, 1.54)	9.1E-24	0.16	0.37	0.17	1.18 (1.07, 1.3)	5.88E-04	0.2827
rs3793917	*ARMS2*	10	124,209,265	G	0.20	0.36	1.43 (1.34, 1.54)	4.3E-24	0.16	0.39	0.17	1.18 (1.07, 1.3)	5.46E-04	0.2944
rs10468017	*LIPC upstr*	15	56,465,804	C	0.70	0.06	0.95 (0.89, 1.01)	0.083	0.12	0.82	0.06	1.06 (0.9, 1.26)	4.58E-01	0.5015
rs493258	*LIPC upstr*	15	56,475,172	C	0.53	0.02	0.98 (0.93, 1.04)	0.581	0.04	0.28	0.15	1.16 (1.03, 1.31)	1.21E-02	0.1815
rs3764261	*CETP*	16	55,550,825	a	0.33	0.07	1.07 (1.01, 1.14)	0.021	0.94	0.25	0.16	1.17 (1.02, 1.35)	2.47E-02	0.4247
rs9621532	*TIMP3*	22	31,414,511	a	0.94	0.05	1.05 (0.92, 1.20)	0.445	0.16	0.95	0.41	1.49 (0.79, 2.81)	2.07E-01	0.4174

a Frequency of effective allele in European-ancestry samples.

b Frequency of effective allele in Singapore-based samples.

*Notes*: Results for SNPs rs9332739, rs9380272, rs641153, rs1999930 and rs2230199 were not tabulated due to a lack of data in the Singapore-based samples (data was available for neither, or only one Singapore-based sample).

## Discussion

We report herein findings from GWAS meta-analysis restricted to individuals with early AMD, prior to the development of late, vision-impairing disease. As expected, SNPs at the *CFH* and *ARMS2/HTRA1* loci, which were discovered for late AMD, comprised strong and significant risk factors for the development of early AMD as well. However, these variants were shown to impart a 3–4 times significantly weaker risk for early than late AMD. Similar effect size ratios for early versus late AMD were observed for additional risk variants in *C2, CFB*, *C3* and *CETP*, with the difference reaching significance for a SNP in *C2*/*CFB*. These findings are consistent with those of studies showing that risk variants in *CFH*, *ARMS2*, *C2*, *CFB* and *C3* influence not only risk of late AMD (based on comparison with normal subjects), but also risk of progression from early to late stages of disease (based on comparison with early AMD cases that did not progress) [32], [33], [34], [35]. A possible explanation for these findings is that the estimated genetic effect size for late AMD comprises cumulative effects of an initial development of early AMD signs and progression from early to late stages of the disease.

It is noteworthy that our large study offered similar sample size to assess genetic association with early AMD to a number of previous GWAS that assessed the associations with late AMD [10], [11], [13], [14], [15], but in contrast to other studies, we detected no significant variants besides *CFH* and *ARMS2* variants at genome-wide significance. This may suggest weaker genetic effects for early AMD, and a possible absence of large effect loci unique to early AMD.

We speculate that the genetic component of early AMD may include risk variants both shared with late AMD, unique to early AMD, and perhaps unique to specific lesions classified here altogether as early AMD. Many confirmed AMD risk variants appear to confer both modest risk of early AMD and late AMD; the presence of these variants may encourage both early disease incidence and steady disease progression throughout time. Additionally, we found suggestive evidence for possible early AMD variants in several novel and biologically plausible genes not previously associated with late AMD. These variants (e.g. within *TYR*, *GLI3* and upstream of *GLI2*) may influence only the initial development of early pathologic features of AMD such as RPE abnormalities and large soft drusen, with alternative pathways mediating disease progression once early stage changes have accumulated.

The GLI3 and GLI2 transcription factors are critical mediators of Sonic Hedgehog (Shh) protein signalling, with species-conserved roles in a range of developmental and adult processes [36]. Notably, Shh signalling is required for the development of Muller glial stem cells [37], a retinal progenitor cell (RPC) with both proliferative and neurogenic ability [38]. Although retinal regeneration has not yet been demonstrated in the adult human, in fish and amphibians acute retinal damage stimulates RPCs of the ciliary marginal zone (CMZ) to proliferate and fully regenerate the multilayered retina throughout life [39](24). Muller glia isolated from the adult human retinal marginal region can also re-enter the cell cycle and demonstrate proliferative potential *in vitro* [38]. The presence of a Muller cell-specific regulatory region upstream of the *HTRA1* promoter [40], and the upregulation of glial fibrillary acidic protein (GFAP) immunoreactivity in the Müller cells of donor retina with drusen [41] may be considered supporting evidence for the involvement of Muller glia in early stages of AMD. It is also interesting to note that GLI3 and GLI2 are zinc finger proteins [42], as zinc supplements have been shown reduce the risk of progression from early to late AMD [43].

We also detected possible association of multiple variants in the *TYR* gene encoding tyrosinase, the enzyme catalysing the first step of melanin biosynthesis. Melanin is produced within pigmented cells including skin melanocytes and retinal pigment epithelium (RPE) cells [44] and deficiencies of melanin in RPE cells have been associated with AMD [44], [45]. Variants in *TYR*, including SNPs correlated with our lead SNP, have previously shown strong association with skin, hair and eye colour [46], [47], tanning ability [48], vitiligo [49] and melanoma risk [50], [51] via GWAS. In addition to protecting from sunlight-induced damage [52], melanin is also an efficient antioxidant, reducing the oxidative stress resulting from lipid peroxidation and reactive oxygen species (ROS) generation [53]. Specifically, elevated melanin content in RPE cells has been shown to reduce accretion of the auto-oxidant lipofuscin [54], a key waste product of photoreceptor outer segment phagocytosis implicated in extracellular drusen formation [3]. This antioxidant function of melanin is consistent with evidence supporting supplemental lutein and zeaxanthin in modifying the course of AMD [55].

The detection of suggestively associated SNPs in the vicinity of the Apolipoprotein E gene (*ApoE*) is not novel, but is consistent with early implication of *ApoE* polymorphisms in AMD [56], [57], [58], [59], [60]. Our most strongly associated SNPs have also previously shown unequivocal association with Alzheimer’s disease diagnosis, pathologic features and biomarkers [61], [62], [63], [64], [65], as well as lipid levels and cardiovascular traits [66], [67] in GWAS.

A potential limitation of this study is possible measurement error in early AMD ascertainment. The milder nature of early AMD signs could result in misclassification of some controls, particularly in the two studies (ARIC and CHS) that examined only non-stereoscopic color retinal photographs of one eye per subject, using non-mydriatic cameras. An estimated misclassification rate for cases and controls could be around 10–20% if using data from one eye per subject. Misclassification would be expected to bias genetic effect estimates for early AMD towards the null, and thus increase the apparent effect differences between early and late AMD. We note though, that the majority of participating cohorts in our study examined stereoscopic retinal photographs of both eyes per subject. Inter-center grading reliability of early AMD has been assessed in a different study across three grading centres (the Wisconsin, BMES and RS) based on stereoscopic images, and showed, for example, 70.2% exact agreement for a 5 step AMD severity scale, or 90.4% agreement if allowing 1 step difference between Wisconsin and BMES graders (personal communication with R.Klein). Furthermore, in sensitivity analyses *excluding* the ARIC and CHS cohorts that used non-stereoscopic photographs of one eye per subject, very little difference in effect estimates was observed for the selected candidate SNPs. In fact, effect estimates tended to be slightly smaller than in the full European meta-analysis (the mean difference in regression coefficients was -0.012, with a standard deviation of only 0.025). If measurement error was a major contributor to the observed effect size differences between early and late AMD, we would have expected the opposite result, i.e. larger effect sizes after reducing measurement error. In addition, a recent Age-relate Eye Disease Study (AREDS) report involving longitudinally assessment of stereoscopic images of AMD patients also documented differential effects of different AMD candidate SNPs on various stages of AMD (from normal to early and then late AMD) [35]. Taken together, this suggests that misclassification error is not an important contributor to the observed effect size differences between early and late AMD detected in this study.

We also acknowledge the potential influence of increased genetic and phenotypic heterogeneity of early AMD, compared to late AMD. If the phenotypic signs of early AMD have a more complex genetic basis, including a larger number of contributing genetic variants, it is possible that a particular AMD risk variant may act in a smaller proportion of subjects than in late AMD. Under this scenario, the effect size in the relevant early AMD subset may be similar to the effect upon late AMD, but the sample-wide estimate will be reduced. Genetic heterogeneity may partially result from phenotypic heterogeneity, if alternative biological processes lead to a clinical presentation that appears as early AMD, but does not progress to late AMD. Although very difficult to quantify or control, such genetic heterogeneity may have contributed to our observed effect estimate differences.

Finally, our analyses of late AMD had limited power, resulting in wide confidence intervals for effect estimates and reducing power to detect effect size differences between early and late AMD. We note however, that analogous comparisons using published estimates from well-powered GWAS of late AMD produced strikingly similar results. As expected, numerous additional SNPs showed significant effect size differences between early and late AMD, but the general pattern of several-fold increased effect sizes for late AMD was clearly consistent with results of the direct internal comparison conducted in our study.

In conclusion, the results of our study confirm the involvement of several established late AMD risk variants in early AMD and provide additional, suggestive evidence for possible risk variants and biological pathways specific to early AMD. These include *TYR* SNPs previously associated with skin and eye pigmentation, and variants in and upstream of *GLI3* and *GLI2*, respectively, potentially influencing retinal regeneration following injury. Our study also demonstrates that many established late AMD genetic risk variants showed reduced effects on early AMD compared to late AMD. Further research should seek to clarify the underlying biological processes involved in early AMD, potentially uncovering novel preventative therapies to prevent the progression of early to late, vision-threatening stages of AMD.

### Web Resources

ProbABEL program: http://mga.bionet.nsc.ru/~yurii/ABEL/

mach2dat software: http://www.sph.umich.edu/csg/yli/mach/index.html

METAL software: http://www.sph.umich.edu/csg/abecasis/Metal/index.html

R software: http://www.r-project.org

(NHGRI) Catalog of Published Genome-Wide Association Studies: http://www.genome.gov/gwastudies/

## Supporting Information

File S1 Supplementary Figures S1– S5.
(DOC)

File S2 Supplementary Tables S1– S3.
(DOC)

File S3 Supplementary Tables S4– S6.
(XLS)
