# Supplementary material for: Insights into the Genetic Architecture of Early Stage Age-Related Macular Degeneration: A Genome-Wide Association Study Meta-Analysis
Source: PLoS One. 2013 Jan 11;8(1):e53830. doi: 10.1371/journal.pone.0053830 (PMC3543264; doi:10.1371/journal.pone.0053830)
Supplement: File S1 — Supplementary Figures S1– S5. (DOC) [file pone.0053830.s001.doc]

Insights into the Genetic Architecture of Early Stage Age-Related Macular Degeneration: a Genome-wide Association Study Meta-analysis

**Supplementary Figures**


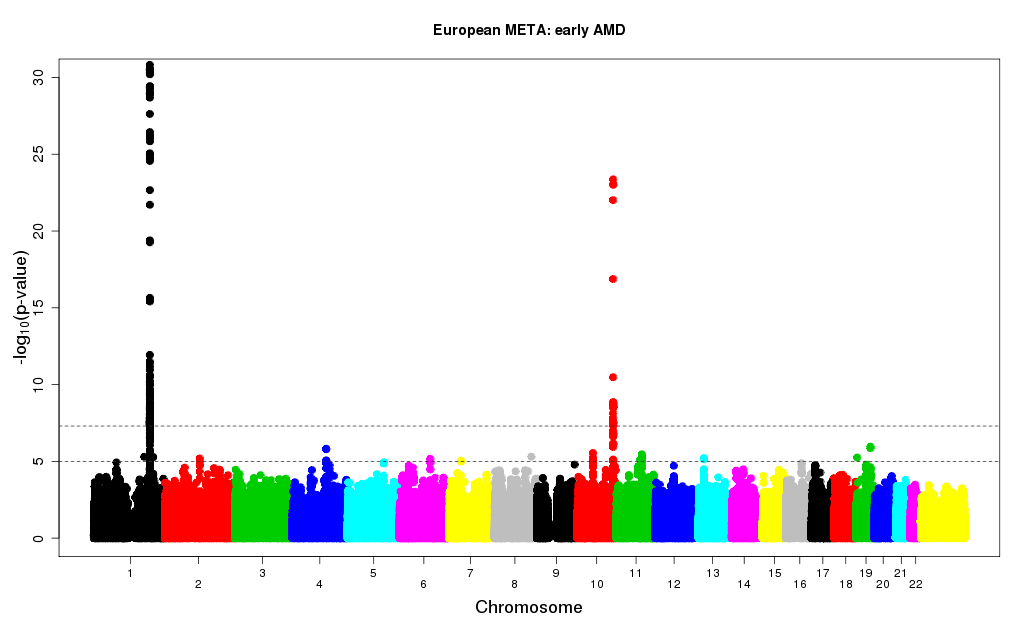


**Figure S1**. Genome-wide association results from the meta-analysis of early AMD in European-Ancestry cohorts (3,772 cases and 16,033 controls). The plots show –log_10_-transformed *P*-values for genotyped and imputed SNPs with respect to their physical position. The threshold for genome-wide significant association (*P*=5×10^-8^) is shown as the upper dashed line. The lower dashed line shows the threshold for suggestive association (*P*=1x10^-5^).


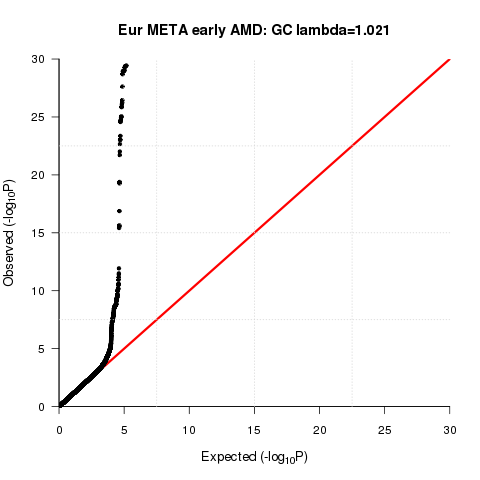


**Figure S2**. Quantile-quantile plot comparing observed and expected –log_10_-transformed *P*-values for GWAS meta-analysis of early AMD in European-ancestry cohorts. The genomic control inflation factor (GC lambda) is shown.


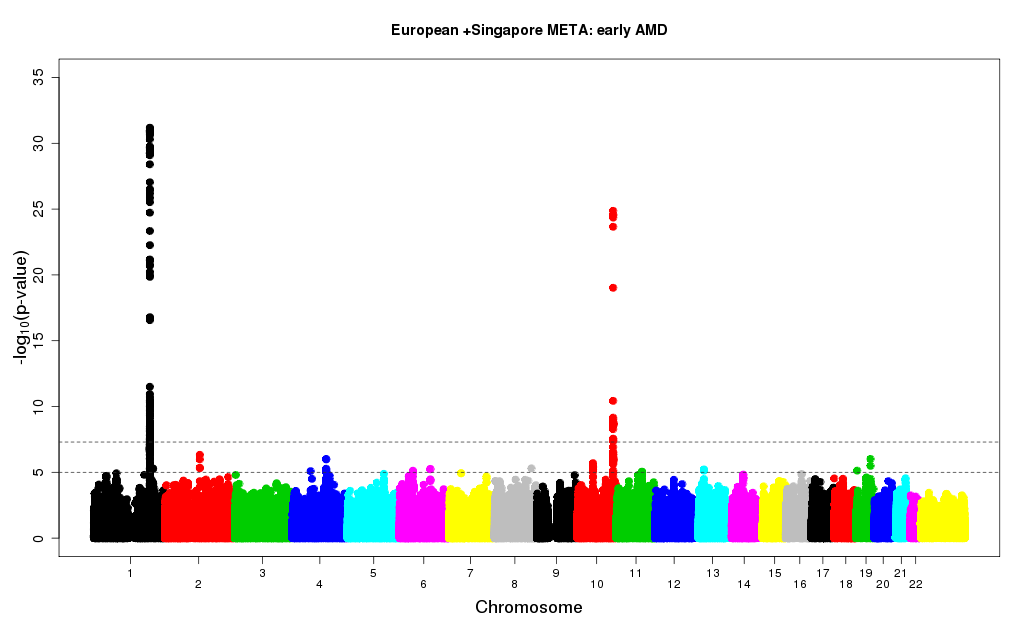


**Figure S3**. Genome-wide association results from the meta-analysis of early AMD in European-Ancestry and Singapore-ancestry combined cohorts (4,036 cases and 19,959 controls). The plots show –log_10_-transformed *P*-values for genotyped and imputed SNPs with respect to their physical position. The threshold for genome-wide significant association (*P*=5×10^-8^) is shown as the upper dashed line. The lower dashed line shows the threshold for suggestive association (*P*=1x10^-5^).


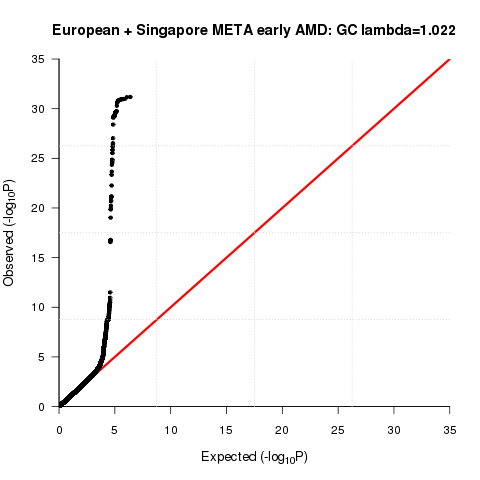


**Figure S4**. Quantile-quantile plot comparing observed and expected –log_10_-transformed *P*-values for GWAS meta-analysis of early AMD in European-ancestry and Singapore-ancestry combined cohorts. The genomic control inflation factor (GC lambda) is shown.


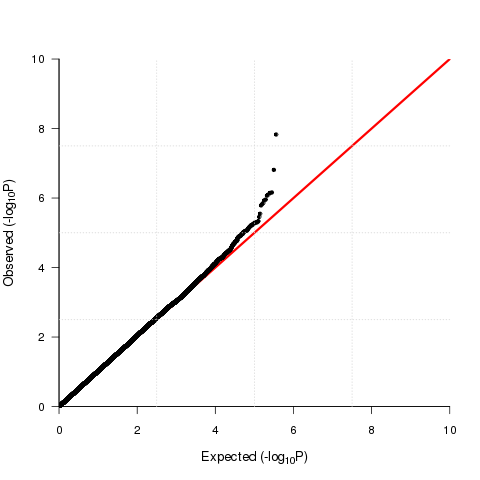

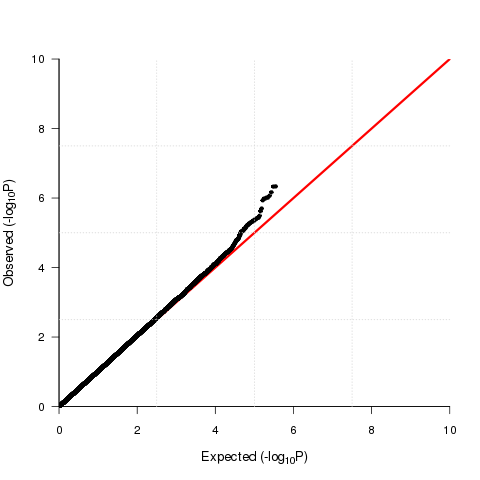


**a**

**b**

**Figure S5**. Quantile-quantile plots for a) European-ancestry and b) European-ancestry and Singapore-ancestry combined cohorts after removing SNPs in the *CFH* and *ARMS2*/*HTRA1* loci.
