# Supplementary material for: Insights into the Genetic Architecture of Early Stage Age-Related Macular Degeneration: A Genome-Wide Association Study Meta-Analysis
Source: PLoS One. 2013 Jan 11;8(1):e53830. doi: 10.1371/journal.pone.0053830 (PMC3543264; doi:10.1371/journal.pone.0053830)
Supplement: File S2 — Supplementary Tables S1– S3. (DOC) [file pone.0053830.s002.doc]

Insights into the Genetic Architecture of Early Stage Age-Related Macular Degeneration: a Genome-wide Association Study Meta-analysis

**Table S1**. Cohort locations and AMD grading details

| **Cohort** | **Location** | **Photography** | **AMD grading scheme** |
| --- | --- | --- | --- |
| AGES^1^ | Reykjavik, Iceland | Stereoscopic retinal images centered on the macula and optic disc using a 6.3 megapixel Canon CR6 non-mydriatic camera with a Canon D-60 camera back (Canon USA, Inc. Lake Success NY). | Masked grading using standardized protocol, at the Ocular Epidemiology Reading Center in Madison, WI |
| ARIC^2^ | Four US communities: Forsyth County, North Carolina; Jackson, Mississippi; suburbs of Minneapolis, Minnesota; Washington County, Maryland | 45° non-stereoscopic color retinal photograph of one eye using a fundus camera that does not require pharmacologic dilation of the pupil (Canon CR-45UAF; Canon USA, Inc., Lake Success, NY). | Masked grading using standardized protocol at the Fundus Photograph Reading Center, University of Wisconsin, Madison, WI |
| BMES^3^ | Blue Mountains region, west of Sydney, Australia | 30º stereoscopic retinal photographs of the macula and other retinal fields of both eyes, using a Zeiss FF3 fundus camera (Carl Zeiss, Oberkochen, Germany). | Masked grading using modified Wisconsin ARM classification scheme |
| CHS^4^ | Four field centers in the USA | Non-stereoscopic color retinal photograph of one eye using a fundus camera. | Masked grading using modified Wisconsin ARM classification scheme |
| RS^5^ | Rotterdam, the Netherlands | After pharmacological mydriasis, full eye examination including stereoscopic fundus photography covering a 35° field centered on the macula (Topcon TRV-50VT fundus camera, Topcon Optical Co, Tokyo, Japan). | Masked grading using modified Wisconsin ARM classification scheme |
| SiMES | Singapore (Malay) | 30º stereoscopic retinal photographs of the macula and other retinal fields of both eyes, using a Zeiss FF3 fundus camera (Carl Zeiss, Oberkochen, Germany). | Masked grading using modified Wisconsin ARM classification scheme |
| SINDI | Singapore (Indian) | Stereoscopic fundus photography using a digital non-mydriatic retinal camera (Canon CR-DGi with a 20Diopter SLR backing, Canon, Japan) using Early Treatment for Diabetic Retinopathy Study (ETDRS) standard field 1 (centered on the optic disc) and ETDRS standard field 2 (centered on the fovea). | Masked grading using modified Wisconsin ARM classification scheme |

^1^ Age Gene/Environment Susceptibility – Reykjavik Study; ^2^ Atherosclerosis Risk in Communities Study; ^3^ Blue Mountains Eye Study; ^4^ Cardiovascular Health Study; ^5^ Rotterdam Study

**Table S2**. Genotyping, imputation and quality control details for individual cohorts

| **Cohort** | **Genotyping array** | **SNP exclusions** | **Sample exclusions** | **Imputation software** | **Imputation reference** |
| --- | --- | --- | --- | --- | --- |
| AGES^1^ | Illumina 370CNV | call rate <0.97, MAF <0.01, HW P <1x10^-6^ | Sample failure, gender discordance, non-European ancestry | MACH | Hapmap II CEU, release 22 NCBI B36 |
| ARIC^2^ | Affymetrix SNP Array 6.0 | call rate <0.90, HW P < 1x10^-6^ | Call rate <0.95, gender discordance, cryptic relatedness (1st degree relatives), non-European ancestry | MACH | Hapmap II CEU, release 22 NCBI B36 |
| BMES^3^ | Illumina Human 670-Quadv1 | call rate <0.95, MAF <0.01, HW P < 1x10^-6^ | Call rate <0.95, gender discordance, outlying autosomal heterozygosity, cryptic relatedness, non-European ancestry | MACH | Hapmap II CEU, release 24 NCBI B36 |
| CHS^4^ | Illumina 370CNV | call rate <0.97, heterozygote frequency=0 , HW P < 1x10^-5^ | Call rate <0.95, gender discordance, non-European ancestry | BIMBAM v0.99 | Hapmap II CEU, release 22 NCBI B36 |
| RS^5^ | Illumina HumanHap550K and 610Q arrays | call rate <0.98, HW P < 1x10^-6^ | Call rate <0.975, outlying autosomal heterozygosity, gender discordance, non-European ancestry | MACH | Hapmap II CEU, release 22 NCBI B36 |
| SiMES | Illumina Human610-Quad | call rate <0.95, monomorphic SNPs, HW P < 1x10^-6^ | Call rate <0.95, cryptic relatedness | IMPUTE v0.5.0 | Hapmap II CHB, JPT, CEU and YRI combined |
| SINDI | Illumina Human610-Quad | call rate <0.95, monomorphic SNPs, HW P < 1x10^-6^ | Call rate <0.95, cryptic relatedness | IMPUTE v0.5.0 | Hapmap II CHB, JPT, CEU and YRI combined |

^1^ Age Gene/Environment Susceptibility – Reykjavik Study; ^2^ Atherosclerosis Risk in Communities Study; ^3^ Blue Mountains Eye Study; ^4^ Cardiovascular Health Study; ^5^ Rotterdam Study

**Table S3**. Membership of Wellcome Trust Case Control Consortium 2

| **Group** | **Members** |
| --- | --- |
| *Management Committee* | Peter Donnelly (Chair)^1,2^, Ines Barroso (Deputy Chair)^3^, Jenefer M Blackwell^4, 5^, Elvira Bramon^6^ , Matthew A Brown^7^ , Juan P Casas^8^ , Aiden Corvin^9^, Panos Deloukas^3^, Audrey Duncanson^10^, Janusz Jankowski^11^, Hugh S Markus^12^, Christopher G Mathew^13^, Colin NA Palmer^14^, Robert Plomin^15^, Anna Rautanen^1^, Stephen J Sawcer^16^, Richard C Trembath^13^, Ananth C Viswanathan^17^, Nicholas W Wood^18^ |
| *Data and Analysis Group* | Chris C A Spencer^1^, Gavin Band^1^, Céline Bellenguez^1^, Colin Freeman^1^, Garrett Hellenthal^1^, Eleni Giannoulatou^1^, Matti Pirinen^1^, Richard Pearson^1^, Amy Strange^1^, Zhan Su^1^, Damjan Vukcevic^1^, Peter Donnelly^1,2^ |
| *DNA, Genotyping, Data QC and Informatics Group* | Cordelia Langford^3^, Sarah E Hunt^3^, Sarah Edkins^3^, Rhian Gwilliam^3^, Hannah Blackburn^3^, Suzannah J Bumpstead^3^, Serge Dronov^3^, Matthew Gillman^3^, Emma Gray^3^, Naomi Hammond^3^, Alagurevathi Jayakumar^3^, Owen T McCann^3^, Jennifer Liddle^3^, Simon C Potter^3^, Radhi Ravindrarajah^3^, Michelle Ricketts^3^, Matthew Waller^3^, Paul Weston^3^, Sara Widaa^3^, Pamela Whittaker^3^, Ines Barroso^3^, Panos Deloukas^3^ |
| *Publications Committee* | Christopher G Mathew (Chair)^13^, Jenefer M Blackwell^4,5^, Matthew A Brown^7^, Aiden Corvin^9^, Chris C A Spencer^1^ |

1 Wellcome Trust Centre for Human Genetics, University of Oxford, Roosevelt Drive, Oxford OX3 7BN, UK; 2 Dept Statistics, University of Oxford, Oxford OX1 3TG, UK; 3 Wellcome Trust Sanger Institute, Wellcome Trust Genome Campus, Hinxton, Cambridge CB10 1SA, UK; 4 Telethon Institute for Child Health Research, Centre for Child Health Research, University of Western Australia, 100 Roberts Road, Subiaco, Western Australia 6008; 5 Cambridge Institute for Medical Research, University of Cambridge School of Clinical Medicine, Cambridge CB2 0XY, UK; 6 Department of Psychosis Studies, NIHR Biomedical Research Centre for Mental Health at the Institute of Psychiatry, King’s College London and The South London and Maudsley NHS Foundation Trust, Denmark Hill, London SE5 8AF, UK; 7 University of Queensland Diamantina Institute, Brisbane, Queensland, Australia; 8 Dept Epidemiology and Population Health, London School of Hygiene and Tropical Medicine, London WC1E 7HT and Dept Epidemiology and Public Health, University College London WC1E 6BT, UK; 9 Neuropsychiatric Genetics Research Group, Institute of Molecular Medicine, Trinity College Dublin, Dublin 2, Eire; 10 Molecular and Physiological Sciences, The Wellcome Trust, London NW1 2BE; 11 Department of Oncology, Old Road Campus, University of Oxford, Oxford OX3 7DQ, UK , Digestive Diseases Centre, Leicester Royal Infirmary, Leicester LE7 7HH, UK and Centre for Digestive Diseases, Queen Mary University of London, London E1 2AD, UK; 12 Stroke and Dementia Research Centre, St George's University of London, London, United Kingdom; 13 King’s College London Dept Medical and Molecular Genetics, King’s Health Partners, Guy’s Hospital, London SE1 9RT, UK; 14 Biomedical Research Centre, Ninewells Hospital and Medical School, Dundee DD1 9SY, UK; 15 King’s College London Social, Genetic and Developmental Psychiatry Centre, Institute of Psychiatry, Denmark Hill, London SE5 8AF, UK; 16 University of Cambridge Dept Clinical Neurosciences, Addenbrooke’s Hospital, Cambridge CB2 0QQ, UK; 17 NIHR Biomedical Research Centre for Ophthalmology, Moorfields Eye Hospital NHS Foundation Trust and UCL Institute of Ophthalmology, London EC1V 2PD, UK; 18 Dept Molecular Neuroscience, Institute of Neurology, Queen Square, London WC1N 3BG, UK.
